# High CD142 Level Marks Tumor-Promoting Fibroblasts with Targeting Potential in Colorectal Cancer

**DOI:** 10.3390/ijms241411585

**Published:** 2023-07-18

**Authors:** András Áron Soós, Andrea Kelemen, Adrián Orosz, Zsuzsanna Szvicsek, Tamás Tölgyes, Kristóf Dede, Attila Bursics, Zoltán Wiener

**Affiliations:** 1 Department of Genetics, Cell and Immunobiology, Semmelweis University, H-1089 Budapest, Hungary; soos.andras@phd.semmelweis.hu (A.Á.S.); kelemen.andrea93@gmail.com (A.K.); oroszadrian77@gmail.com (A.O.); zsuzsanna.szvicsek@gmail.com (Z.S.); 2 Uzsoki Teaching Hospital, H-1145 Budapest, Hungary; tolgyestamas5@gmail.com (T.T.); dede.kristof@gmail.com (K.D.); bursics@uzsoki.hu (A.B.)

**Keywords:** colorectal cancer, cancer-associated fibroblast, CAF, organoid, drug sensitivity, heterogeneity, inhibitor, BCL family, hepatocyte growth factor, HGF, tissue factor, F3, HSP90, JQ1, MEK

## Abstract

Colorectal cancer (CRC) has a high incidence and is one of the leading causes of cancer-related death. The accumulation of cancer-associated fibroblasts (CAF) induces an aggressive, stem-like phenotype in tumor cells, and it indicates a poor prognosis. However, cellular heterogeneity among CAFs and the targeting of both stromal and CRC cells are not yet well resolved. Here, we identified CD142^high^ fibroblasts with a higher stimulating effect on CRC cell proliferation via secreting more hepatocyte growth factor (HGF) compared to CD142^low^ CAFs. We also found that combinations of inhibitors that had either a promising effect in other cancer types or are more active in CRC compared to normal colonic epithelium acted synergistically in CRC cells. Importantly, heat shock protein 90 (HSP90) inhibitor selected against CD142^high^ fibroblasts, and both CRC cells and CAFs were sensitive to a BCL-xL inhibitor. However, targeting mitogen-activated protein kinase kinase (MEK) was ineffective in fibroblasts, and an epigenetic inhibitor selected for a tumor cell population with markers of aggressive behavior. Thus, we suggest BCL-xL and HSP90 inhibitors to eliminate cancer cells and decrease the tumor-promoting CD142^high^ CAF population. This may be the basis of a strategy to target both CRC cells and stromal fibroblasts, resulting in the inhibition of tumor relapse.

## 1. Introduction

Colorectal cancer (CRC) is one of the most prevalent cancer types and a leading cause of cancer-related deaths in developed countries. APC inactivation is a common initializing mutation in CRC tumorigenesis, leading to the continuous and unregulated activation of the Wnt pathway. This results in the uncontrolled proliferation of the intestinal epithelial cells and in adenoma formation. Some of these adenomas accumulate further mutations in driver genes such as *KRAS*, *SMAD4*, and *TP53*, and they progress to invasive carcinomas. Importantly, tumors contain cells with varying mutational and gene expression profiles, resulting in intra-tumoral cellular heterogeneity that may also lead to phenotypic variation. Since patient-derived organoids may represent the genetic and cellular heterogeneity of in vivo epithelial tumors when cultured in 3D matrices under well-defined conditions, they serve as a tool for studying cellular plasticity and for carrying out drug screenings [1,2].

The accumulation of cancer-associated fibroblasts (CAF), an important and abundant cell type in the stroma, results in a worse patient survival in CRC [3,4]. Importantly, fibroblasts provide a niche for CRC cells with stem-like phenotypes and aggressive features, e.g., by secreting hepatocyte growth factor (HGF) or osteopontin [5,6]. In addition, the recent CRC classification system based on gene expression profiling resulted in four subgroups (consensus molecular subtype, CMS1-4) where CMS4 displays fibroblast accumulation and epithelial-mesenchymal transition (EMT), and has the worst survival rate [7]. Interestingly, an inflammatory CAF subtype has been identified in pancreatic ductal adenocarcinoma (PDAC), a cancer type with an extremely low survival rate and a high fibroblast content [8,9]. Whereas the myofibroblastic fibroblasts (myCAFs) produce primarily extracellular matrix (ECM) components, such as collagen, the other CAFs acquire inflammatory properties (iCAFs) and secrete inflammatory cytokines [8,9]. Importantly, the iCAF phenotype is induced by IL-1α [9]. Similarly, the presence of myCAF and iCAF populations has recently been suggested in CRC as well [10]. Furthermore, single-cell RNA sequencing experiments have identified CAF-A and CAF-B. Whereas CAF-B cells expressed markers of myofibroblasts, these genes were downregulated in CAF-A cells [11]. Another study classified CAFs into the adhesion/wound healing and the perivascular subtypes. Of note, the former population could be further divided into myCAFs, characterized by the enhanced expression of collagen-related genes and fibroblast markers, and into iCAFs, producing chemokines [12]. Interestingly, a more detailed analysis allowed the identification of further subgroups [12]. In addition, a specific CAF subpopulation can induce EMT in CRC cells via Wnt activation, leading to an aggressive phenotype of CRC [13]. Collectively, despite the importance of CAFs in CRC progression and patient survival, the classification of fibroblasts and CAFs is still not fully resolved in CRC.

Thus, characterizing stromal fibroblast heterogeneity and therapeutic targeting of both cancer and stromal cells in CRC are major challenges. In a study focusing on normal and inflammatory colons, the authors found four basic fibroblast populations with characteristic gene expression profiles and functions using single cell sequencing. Importantly, the size of one of these subpopulations largely increased in inflammatory bowel disease and these fibroblasts were critically involved in maintaining inflammatory processes. These cells expressed CD142 at a low and podoplanin (PDPN) at a high level [14]. In contrast to the massively increased number of PDPN+ fibroblasts, there was a decrease in the size of CD142+ fibroblast population in colitis [14]. CD142, also named tissue factor, factor III, or thromboplastin, is a transmembrane glycoprotein that is essential for the coagulation cascade. When coagulation factor VII binds to CD142, it is converted to the active form factor VIIa, which in turn stimulates the activation of factors IX and X. Besides this function, and depending on the cellular context, CD142 may induce mitogen activated protein kinase (MAPK), phosphatidyl-inositol-3-kinase (PI3K), or Wnt signaling in cancers [15].

Thus, we hypothesized that differential expression of CD142 or PDPN may mark CAFs with differential roles in CRC tumorigenesis. We then also tested novel compound combinations on CRC cells and fibroblasts, and we compared their effects on the tumor supporting fibroblast subpopulation.

## 2. Results

### 2.1. CD142^high^ Stromal Cells Are Present in CRC

**Figure 1:**
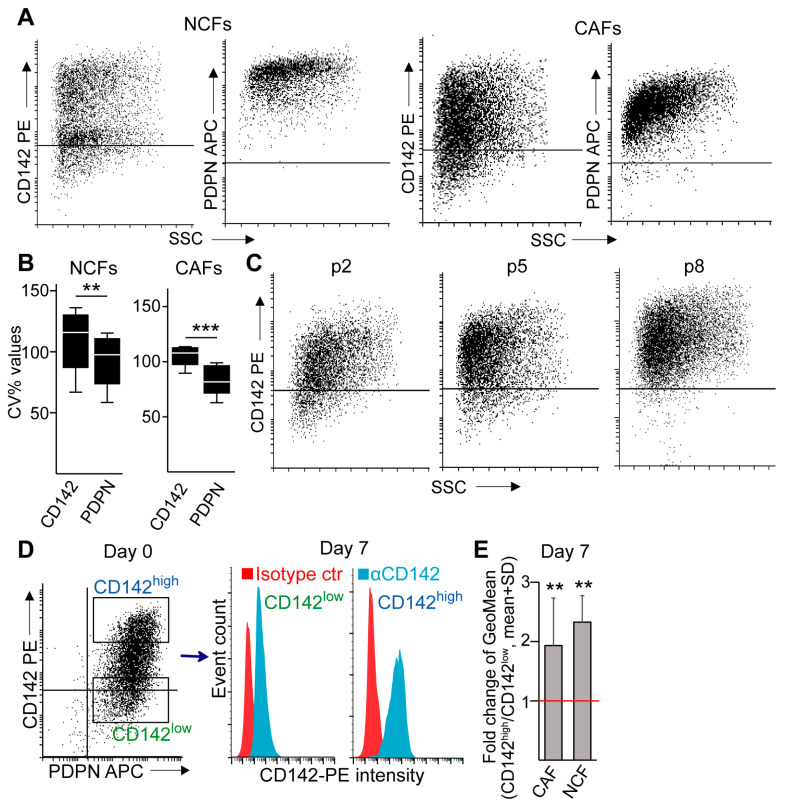
Fibroblasts show a heterogeneity for CD142. (**A**) PDPN and CD142 expression in normal colon fibroblasts (NCF) and tumor-derived fibroblasts (CAF). The horizontal line indicates the fluorescence intensity of the isotype control (representative flow cytometry images). (**B**) Coefficient of variation for the geometric mean values for CD142 and PDPN from flow cytometry analysis (n = 8 for CAF and n = 4 for NCF). Note the high CV value for CD142 for both fibroblast types. (**C**) Representative images from CAF cultures at different time points after isolation from patients (flow cytometry, p: passage number). (**D**) Sorting strategy for CD142^high^ and CD142^low^ cells (left panel), and CD142 detection on the sorted cells after 7 days in culture (flow cytometry). (**E**) The ratio of CD142 fluorescence intensity geometric mean (GeoMean) values derived from CD142^high^ and CD142^low^ sorted CAFs and NCFs after 7 days in culture (flow cytometry, n = 11 for CAF and n = 3 for NCF). The red line indicates no difference between the two populations (ratio = 1). Mann–Whitney U-test (**B**) or paired *t*-test (**E**) were used with ** *p* < 0.01, *** *p* < 0.005.

Heterogeneity within CAFs is critical in the initialization and progression of CRC, and CD142 and PDPN may show different fibroblast subpopulations. To test these two markers, we first analyzed the Protein Atlas Database (www.proteinatlas.org, accessed on 28 April 2023) containing quantitative immunostaining data. This indicated that tumor cells had only a low and moderate level of CD142 and PDPN, respectively (Figure S1A). To test the stromal expression of these molecules, we isolated fibroblasts from CRC patient tumor tissues (CAFs). Importantly, these cultures expressed the mesenchymal markers *ACTA2* (encoding for αSMA) and fibroblast activation protein (*FAP*), confirming the fibroblast identity of the cells (Figure S1B,C). In addition, we could not detect the epithelial markers *CDH1* and *EpCAM* in these CAFs (Figure S1B). Flow cytometry proved the presence of both PDPN and CD142 in all cultures (Figure 1A,B). Although differences in binding properties of the antibodies cannot be ruled out, these CAFs showed a large heterogeneity for CD142 (Figure 1A,B): thus, we focused on this molecule in our subsequent experiments. The heterogeneity for CD142 was maintained during the prolonged culturing of CAFs (Figure 1C). In addition, sorted CD142^high^ and CD142^low^ CAFs and commercially available human normal colon fibroblasts (NCF) maintained the high and low level of this molecule at least for one week in culture (Figure 1D,E). We also observed CD142+ stromal cells, but not tumor cells in CRC patient-derived tissue sections (Figure S1D). Thus, CD142 is present on stromal cells in CRC, and its different level may mark fibroblasts of specific features.

### 2.2. CD142 Stimulation of Fibroblasts Has no Major Role in CRC Cell Proliferation in Co-Cultures

We next used CRC patient-derived 3D organoids that had already been extensively characterized in our previous publications as a test system. Of note, these organoid lines are dependent on external EGF activity [16,17]. As expected, CRC cells had only a low level of surface CD142 (Figure S2A). When removing EGF from the culture medium, we observed a decrease both in the organoid diameter and the ratio of KI67+ proliferating cells (Figure S2B). However, the addition of factor VIIa, which serves as the active ligand of CD142, had no effect on these parameters either in the presence or absence of EGF (Figure S2B). In addition, factor VIIa did not induce the proliferation of CAFs (Figure S2C). When culturing organoids with fibroblasts (Figure S2D), CAFs increased the organoid diameter and the number of KI67+ CRC cells only in the absence of EGF (Figure S2E), suggesting that CAFs may act via growth factors. Again, the addition of factor VIIa to the co-cultures had no effect on cell proliferation and the organoid diameter independently of the presence of EGF (Figure S2E). Collectively, CAFs induced by factor VIIa did not stimulate CRC cell proliferation. Although factor VIIa may be effective only with some other ligands in fibroblasts, these results may also raise the possibility that CD142 is only a marker of specific fibroblast subpopulations.

### 2.3. TGFβ Results in the Accumulation of CD142^high^ Fibroblasts with a Mixed Phenotype of myCAFs and iCAFs in CRC

**Figure 2:**
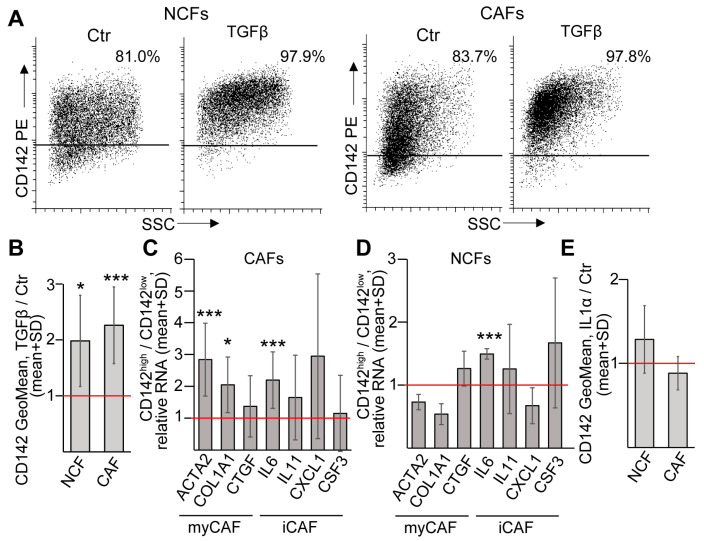
CD142^high^ fibroblasts show a mixed iCAF and myCAF phenotype. (**A**) Representative plots from untreated or TGFβ-treated (10 ng/mL for 4 days) NCFs or CAFs (flow cytometry). (**B**) The ratio of CD142 fluorescence signal intensities (GeoMean) from TGFβ-treated and control NCFs or CAFs (n = 11 for CAFs and n = 7 for NCFs, paired comparisons). The red line indicates no difference (ratio = 1). (**C**,**D**) Comparing the RNA levels of the indicated genes in freshly sorted CD142^high^ and CD142^low^ CAFs (**C**) or NCFs (**D**). RNA levels were normalized with a housekeeping gene (*GAPDH*) and the ratio of these normalized RNA levels were plotted (n = 5–7 for CAFs and n = 3 for NCFs). (**E**) Relative changes in the intensity of cell surface CD142 level (GeoMean) when CAFs or NCFs were cultured in the presence or absence of IL-1α (5 ng/mL for 4 days, n = 11 for CAFs and n = 4 for NCFs). Paired *t*-tests (**B**–**E**) were carried out with * *p* < 0.05, *** *p* < 0.005.

TGFβ is a major inducer of fibroblast activation in CRC [3]. We have previously shown that TGFβ increases the expression level of several genes connected to fibroblast activation (both NCF and CAF) in CRC, such as *ACTA2*, *FAP*, *IL11*, *IL6*, and *HBEGF*, and it induces the appearance of IL6+ cells [18]. To test how TGFβ modifies CD142 expression, we first applied NCFs. Importantly, these fibroblasts were heterogeneous for CD142, and TGFβ resulted in a shift towards the CD142^high^ subpopulation (Figure 2A,B). Similarly, TGFβ induced the accumulation of CD142^high^ cells in CAF cultures, too (Figure 2A,B). These data suggest that CD142 marks activated fibroblasts in CRC.

Since CD142 is characteristic for a fibroblast population in the normal colon that may contribute to epithelial stem cell proliferation and may form a mesenchymal niche [14], this raises the possibility that CD142^high^ marks myCAF-like cells in CRC. Although sorted CD142^high^ CAFs (see Figure 1D) expressed *ACTA2* and *COL1A1* at a higher level than CD142^low^ cells, we found no difference in *CTGF* that is also a myCAF marker (Figure 2C). Interestingly, we also observed an elevated RNA level for the inflammatory gene *IL6*, but not for other molecules characteristic for iCAFs, such as *IL11*, *CSF3*, and *CXCL1* in CD142^high^ fibroblasts (Figure 2C), suggesting that they do not acquire either the myCAF or iCAF phenotype. Similarly, normal colon CD142^high^ fibroblasts differed only in their *IL6* RNA level compared to CD142^low^ cells (Figure 2D). Of note, IL1α, a critical factor for polarizing fibroblasts to iCAFs, had no effect on NCFs or CAFs (Figure 2E). Collectively, these results indicate that CD142^high^ fibroblasts do not show either iCAF or myCAF features; they are in an activated state and TGFβ selects this population and/or converts CD142^low^ cells to CD142^high^ fibroblasts.

### 2.4. CD142^high^ Fibroblasts Increase CRC Organoid Producing Frequency and Cell Proliferation via Secreting HGF

**Figure 3:**
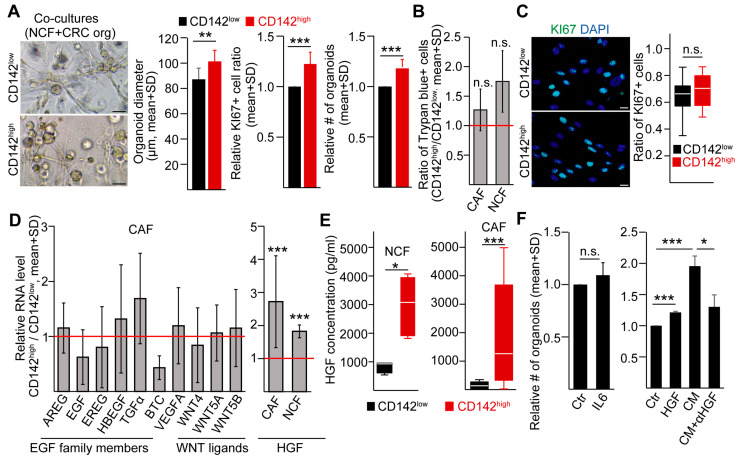
CD142^high^ fibroblasts increase CRC organoid producing frequency and cell proliferation via secretion of HGF. (**A**) CRC organoid diameter, the relative number of KI67+ proliferating CRC cells and the relative number of colonies produced by CRC organoid cells in co-cultures with sorted CD142^high^ or CD142^low^ colon fibroblasts (n = 8, four organoid lines were tested twice). Evaluation was carried out on day 7 after plating. The left panel shows representative light microscopy images (scale bars: 100 µm). (**B**) Trypan blue viability staining (n = 7 for CAFs and n = 4 for NCFs) and (**C**) KI67 immunostaining of sorted fibroblasts on day 7 (n = 7, representative images and quantification, scale bars: 40 µm). For trypan blue staining, samples from the same experiment were compared and the relative ratio is shown. (**D**) Comparing the RNA levels of the indicated genes in sorted CD142^high^ and CD142^low^ CAFs (left panel, n = 3–6) or the RNA level of HGF in CAFs and NCFs (right panel, n = 10 for CAFs and n = 3 for NCFs). RNA levels were normalized to the *GAPDH* housekeeping gene and the ratio of these normalized RNA values were plotted. The red line indicates no difference (ratio = 1). (**E**) HGF protein concentration in the conditioned media of CD142^high^ and CD142^low^ CAFs or normal colon fibroblasts (ELISA, n = 6 for NCF and n = 12 for CAF). Conditioned medium samples were collected from confluent cultures after 72 h. (**F**) Relative colony forming efficiency of four organoid lines in the presence of the indicated treatments (IL-6 50 ng/mL, HGF 50 ng/mL, αHGF 5 µg/mL for 4 days). Conditioned media (CM) from NCFs were used after 72 h of culturing (n = 4 from four organoid lines). Unpaired *t*-test (**A**, left panel), paired *t*-test (**A**, middle and right panels, **B**,**D**,**F**), or Mann–Whitney U-test (**C**,**E**) were used with * *p* < 0.05, ** *p* < 0.01, *** *p* < 0.005 and n.s.: *p* > 0.05.

To study whether these two fibroblast subpopulations have different effects on CRC cells, we sorted CD142^high^ and CD142^low^ NCFs and co-cultured them with CRC organoid cells as a test system. CD142^high^ fibroblasts increased the organoid forming efficiency, the diameter of organoids and the percentage of KI67+ proliferating cells of organoids compared to CD142^low^ fibroblasts (Figure 3A). However, we found no difference in the ratio of viable cells between these two populations of both NCFs and CAFs (Figure 3B). In addition, CD142^high^ and CD142^low^ CAFs did not differ in the number of proliferating cells (Figure 3C). Thus, fibroblasts with differential CD142 levels have different organoid initializing effects in CRC.

To identify the underlying mechanism, we tested the RNA level of several genes from the epidermal growth factor family members, VEGF, Wnt ligands, and hepatocyte growth factor (HGF) that are known to be secreted by fibroblasts (Figure 3D). Whereas factor VIIa had no effect on *HGF* RNA level in unsorted NCFs or CAFs (Figure S2F), we found a significant difference between CD142^high^ and CD142^low^ fibroblasts in *HGF* expression (Figure 3D), and we confirmed our finding at the protein level with ELISA, too (Figure 3E). In contrast to IL-6, HGF increased the organoid forming efficiency of CRC cells (Figure 3F and Figure S2G). In addition, blocking HGF in fibroblast-derived conditioned medium reduced the number of novel CRC organoids (Figure 3F and Figure S2G). Collectively, these data show that the higher secretion of HGF by CD142^high^ fibroblasts is involved in the differential cancer stimulating effect of these two fibroblast populations.

### 2.5. Synergistic Effects of Novel Drug Combinations in CRC Patient-Derived Organoids

**Figure 4:**
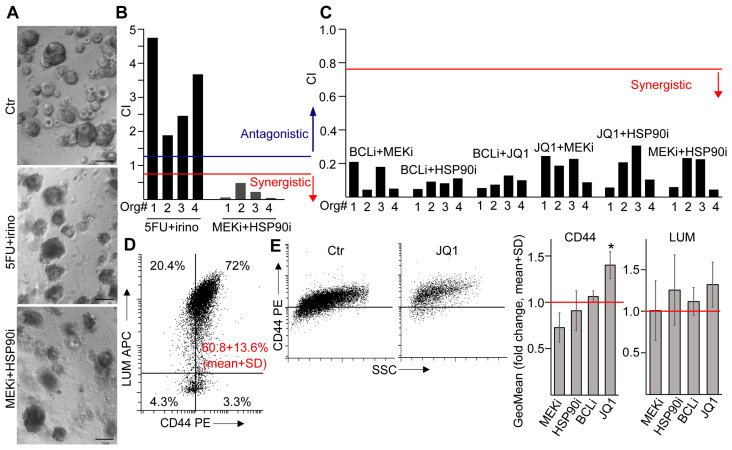
Synergistic effects of combination treatments in CRC patient-derived organoids. (**A**–**C**) Representative light microscopy images of CRC organoids (**A**) and the synergistic or antagonistic effects of the indicated drug combinations (CI: combination index). Note that CI < 0.75 (red line) or CI > 1.25 (blue line) indicate synergistic or antagonistic effects, respectively (see Materials and methods). Images were taken from cultures treated with the IC50 value of the drug combinations (see Table S1) in Matrigel. (**D**) CD44 and LUM levels in CRC organoids (flow cytometry). The red numbers indicate the percentage of double positive cells from the four organoid lines (n = 4 from four organoid lines). (**E**) The effect of treatments on the expression intensity of CD44 or LUM in CRC organoid cells. Representative flow cytometry plot (**left** panel) and the quantitative analysis of the geometric mean (GeoMean) values (**right** panels, n = 4–8). Paired *t*-test (**E**) with * *p* < 0.05. Scale bars: 100 µm.

Since fibroblasts provide a critical niche for CRC cells of aggressive features [5,6], targeting these cells may have a major therapeutic value. To characterize the response of CD142^high^ and CD142^low^ fibroblast subpopulations to drug treatments, we first determined the effect of novel compound combinations on CRC organoids. The combination of the MEK1/2 inhibitor Trametinib (MEKi) and HSP90 inhibitor PU-H71 (HSP90i) has recently been shown to be an effective potential therapy in other cancers without a critical side effect [19]. To test this combination in CRC, we applied our patient-derived 3D organoids that are dependent on external EGF activity and do not carry mutations in *KRAS* [16,17]. We observed a reduced phosphorylation of p42/44 (Erk1/2) and the ribosomal protein S6 after treatment with MEKi and HSP90i (Figure S3A), showing that this drug combination is effective in CRC organoids. In contrast to the traditional therapy combination of 5-fluorouracil (5FU) and irinotecan (irino) where we found an antagonistic effect of the two drugs, MEKi and HSP90i acted synergistically in all the tested organoid lines (Figure 4A,B and Table S1 for IC50 values).

The accumulation of collagen I in the extracellular matrix (ECM) is a general hallmark in CRC progression. As described previously, culturing organoids in collagen I induced the migration of cancer cells (Figure S3B,C and [17,20]). Interestingly, CRC organoids were positive for the epithelial marker EpCAM even in collagen, although some cells had a lower level of this protein compared to Matrigel (Figure S3C,D). At the same time, we observed the overexpression and underexpression of EMT marker genes and Wnt target genes, respectively (Figure S3E), however, collagen had no major effect on the chemosensitivity of the organoids (Figure S3F).

Recently, a large-scale screening found that CRC organoids are more susceptible to the bromodomain epigenetic inhibitor JQ1 compared to wild-type colon organoids [21]. Furthermore, since CRC cells are dependent on BCL-xL, but normal intestinal stem cells are not, the highly potent and selective BCL-xL inhibitor A-1155463 (BCLi) may selectively kill tumor cells [22]. To extend our experiments for more drug combinations, we involved these compounds in our tests. Importantly, out of the tested BCL family members, all CRC organoid lines expressed *BCL-xL* at the highest level (Figure S3G) and the expression pattern of the *BCL* gene family did not change when culturing organoids in collagen I (Figure S3G). In line with these data, BCLi reduced the viability of our organoid lines (Figure S3H). Interestingly, BCLi showed a synergistic effect when combined with JQ1, MEKi, or HSP90i (Figure 4C). Similarly, JQ1 acted synergistically with all other compounds (Figure 4C and Table S1 for IC50 values).

Next we applied the IC50 concentration of the compounds (Table S1) where only a fraction of cells survive, ensuring comparable analysis of the samples. As expected, we observed a reduced proliferation and induced apoptosis for all compounds (Figure S3I). CD44 marks an aggressive tumor-promoting CRC cell population [23] and lumican (LUM) is a mesenchymal marker that is upregulated in CRC cells in collagen cultures during EMT [20]. Interestingly, a large percentage of the CD44+ CRC cells also expressed LUM (Figure 4D) that may represent a high-plasticity cellular state in EMT with both epithelial and mesenchymal markers [24]. Although we found no change in the intensity of LUM expression with either compounds at IC50 concentrations (Figure 4E), we observed an increase in the expression intensity of CD44 for JQ1, but not for the other compounds (Figure 4E). Thus, only JQ1 resulted in a shift towards the CD44^high^ phenotype within the surviving cell population. In addition, BCLi resulted in no major changes in the expression pattern of *BCL* family members in a combination setting (Figure S3J), suggesting the lack of selection for tumor cells with a different *BCL* family expression profile. Collectively, these results indicate that out of the four compounds, only JQ1 has a positive selection on the CD44^high^/LUM+ CRC cell population.

### 2.6. HSP90i Results in the Negative Selection of CD142^high^ Fibroblasts

**Figure 5:**
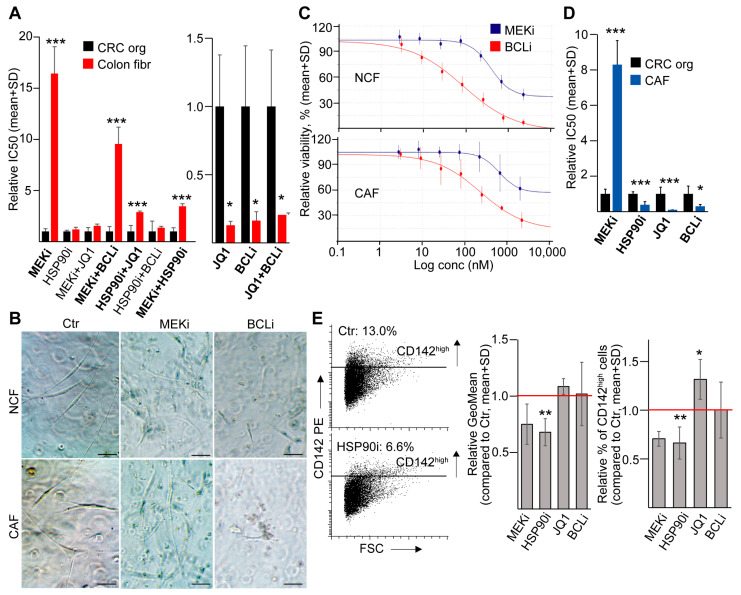
HSP90i results in the negative selection of CD142^high^ fibroblasts. (**A**) Relative IC50 values for CRC organoids and NCFs. For each treatment, the average of organoid IC50 was taken as one for comparison. Treatments with significant IC50 difference between CRC cells and fibroblasts are marked in bold (n = 4 for CRC organoids and n = 3 for fibroblasts). (**B**) Representative light microscopy images from NCFs and CAFs treated with the IC50 concentration (see Table S1) of the indicated compounds in Matrigel. (**C**) Viability curves for NCFs and CAFs treated with an increasing concentration of the MEKi and BCLi. Note that MEKi did not result in a complete killing of fibroblasts even at high concentrations. (**D**) Relative IC50 values from CRC organoids (n = 4) and CAFs (n = 4) for the indicated compounds. (**E**) Relative level of CD142 and the relative percentage of CD142^high^ NCFs (flow cytometry, n = 4). Note that results were compared to the untreated control (red line). The left panel shows representative plots. Unpaired *t*-test (**A**,**D**) or paired *t*-test (**E**) were used with * *p* < 0.05, ** *p* < 0.01, and *** *p* < 0.005. Scale bars: 20 µm.

Having determined the chemosensitivity of CRC organoid cells, we next focused on the effect of these compounds on fibroblasts. NCFs showed a higher resistance for MEKi and MEKi/HSP90i, MEKi/BCLi, and HSP90i/JQ1, but they were more sensitive to BCLi, JQ1, and the combination of these two drugs compared to CRC organoids (Table S1 and Figure 5A,B). Of note, even large doses of MEKi did not result in the complete killing of the fibroblasts (Figure 5C). Importantly, we also confirmed results from the single compounds for CAFs (Figure 5B,D and Table S1). When applying the IC50 concentrations of the drugs, we observed a decreased percentage of CD142^high^ fibroblasts and a reduced level of cell surface CD142 for HSP90i, but not for JQ1 or BCLi (Figure 5E). Collectively, JQ1 selects for a CD44^high^/LUM+ CRC cell population and MEKi does not effectively target fibroblasts that are an important niche cell type of the stroma. Since fibroblasts are sensitive to BCLi, and HSP90i results in a negative selection of the tumor-promoting CD142^high^ fibroblasts, this combination may target both tumor cells and stomal fibroblasts in CRC.

**Figure 6:**
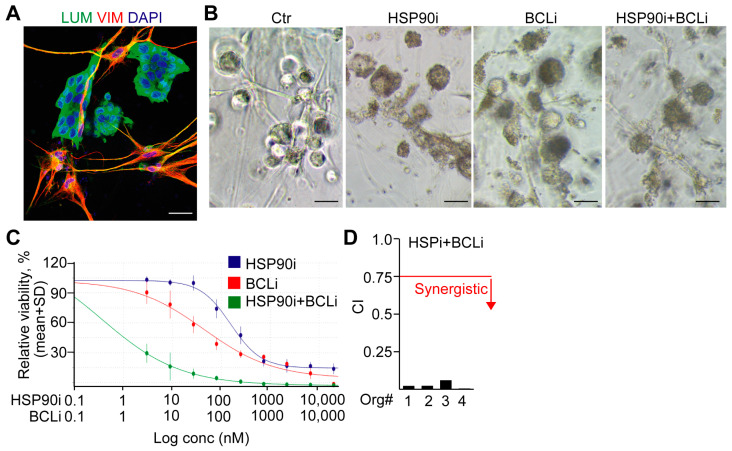
The combination of HSP90i and BCLi has a synergistic effect in co-cultures. (**A**) Immunostaining of co-cultures from NCFs and CRC organoids. (**B**) Representative light microscopy images from co-cultures (NCFs and CRC organoids) treated with the IC50 values of the indicated compounds. (**C**) Representative viability curves from co-cultures with HSP90i, BCLi, or their combination. (**D**) The synergistic effect of HSP90i and BCLi in the co-cultures of NCFs and CRC organoids (CI: combination index, CI < 0.75 indicates synergistic effect, marked with a red line). Scale bars: 40 µm (**A**) or 100 µm (**B**).

To prove that the contact between fibroblasts and CRC cells does not modify the effect of the proposed HSP90i and BCLi combination, we set up co-culture experiments with NCFs and CRC organoid cells, and we imaged them using vimentin and lumican immunostaining, respectively (Figure 6A,B). Of note, the combination of HSP90i and BCLi resulted in a lower IC50 value compared to the single treatments (Figure 6B,C), and we also observed a strong synergy between them in co-cultures (Figure 6D). Thus, these results further indicate that the combination of HSP90i and BCLi may efficiently target both CRC and stromal cells, including CD142^high^ fibroblasts.

## 3. Discussion

By using patient-derived organoid and stromal fibroblast co-cultures, here we identified a tumor-supporting fibroblast population in CRC, marked with increased CD142 level. We also provided evidence that CD142^high^ and CD142^low^ CAFs differed in their HGF secretion, HGF induced the formation of novel organoids, and this molecule is an important factor in the conditioned medium of fibroblasts. All these data provide an explanation for the higher organoid-forming efficiency and CRC cell proliferation intensity induced by CD142^high^ fibroblasts. Moreover, we proved that, whereas both fibroblasts and CRC cells responded to BCL-xL, HSP90, epigenetic inhibitor and their combinations, the bromodomain epigenetic inhibitor JQ1 selected a CD44+/LUM+ CRC cell population. Furthermore, the HSP90 inhibitor resulted in a decrease of CD142^high^ fibroblasts. Thus, the CAF subpopulation and CRC cells may be targeted efficiently by the combination of BCL-xL and HSP90 inhibitors that act synergistically. 

In PDAC, myCAFs produce primarily ECM components and iCAFs secrete inflammatory cytokines [8]. Interestingly, IL-1α and IL-1β can induce the differentiation of iCAFs [9]. During inflammation, CD142 can be upregulated by a variety of cytokines, including interferon-γ (IFNγ), tumor necrosis factor-α (TNFα), IL-6, IL-1β, and IL-33 in different cell types [25,26,27,28,29]. Although iCAFs and myCAFs have been suggested for CRC as well, IL-1α did not have an effect on the CD142 level in our fibroblasts. In addition, we found that CD142^high^ CAFs expressed both iCAF and myCAF markers, suggesting that these cells have a mixed feature. Instead, our data show that TGFβ, a main inducer of fibroblast activation, increased CD142 level in fibroblasts and/or it selected the CD142^high^ population. Importantly, the TGFβ-induced gene expression profile represents a bad prognosis for CRC patients [4]. Furthermore, CD142 expression is associated with staging and metastasis in CRC [15,30]. Of note, the TGFβ-induced CD142 expression is in line with results from Wygrecka et al. in human lung fibroblasts [31]. Furthermore, CD142^high^ breast adipose progenitor cells have a high capacity of differentiation into tumor-promoting myofibrotic CAFs, thus strengthening our findings [32]. Our results suggest that CD142^high^ may mark an activated CAF population. In line with this notion, we and others have previously found that TGFβ increased the RNA level of genes that are characteristic for activated fibroblasts [3,18], and CAF heterogeneity increased in the presence of TGFβ [18].

CD142 is constitutively expressed in the sub-endothelial tissues and adventitia, where it acts as a hemostatic envelope to prevent bleeding when it binds calcium and coagulation factors. In normal embryonic lung fibroblasts, CD142–FVIIa activation upregulated the expression of CCN1, a matrix signaling protein that is a ligand for integrin αvβ3 on endothelial cells. Integrin αvβ3 functions as an adhesion receptor and it regulates a number of cellular processes, including cell adhesion, migration, and tumor metastasis [33]. In addition, cell signaling initiated by CD142–FVIIa has also been shown to promote platelet-derived growth factor BB (PDGF-BB)-induced cell migration in normal skin fibroblasts [34]. CD142–FVIIa cleavage of protease activated receptors (PARs) causes transactivation of multiple receptor tyrosine kinases, including EGF receptor in keratinocytes, PDGF receptor β in monocytes, endothelial cell and fibroblasts, and IGF-1 receptor in breast cancer cells [35,36,37]. Despite these published data, we could not find an effect of factor VIIa on CD142+ fibroblasts when testing the RNA level of several genes and the organoid-forming efficiency of fibroblasts. This suggests that CD142 may only be a marker of a specific fibroblast subpopulation. Alternatively, the activation of CD142 is effective only in the presence of another parallel signal, or it activates mechanisms that may modify tumorigenesis only in the presence of the complete microenvironment. 

We found here that CD142^high^ CAFs have a higher tumorigenic effect compared to CD142^low^ cells. Interestingly, previous publications have proved that CAFs are a rich source of EGF family members, and some of them are transmitted via extracellular vesicles [38,39]. However, we could not find a differential expression in EGF family members when comparing the two fibroblast populations. In contrast, CD142^high^ cells produced more HGF and this molecule was critical in transmitting the proliferation stimulating effect of fibroblasts to CRC organoid cells. In line with our observations, the accumulation of CAFs leads to a poor prognosis in CRC, and CAFs secrete molecules, such as HGF, that induce the aggressive, stem-like phenotype of cancer cells [6]. HGF/c-MET activates multiple cell signaling pathways leading to tumor cell proliferation, such as the RAS, PI3K, or Wnt/β-catenin pathway [40]. Furthermore, CAF-derived HGF has been detected in multiple tumor types as a positive regulator of cancer progression [41,42,43], thus underlining the important role of differential HGF secretion in the stromal-tumor communication.

Cellular plasticity is a central feature of cancers. Organoids are considered as one of the best current tools to model human epithelial tumors when cultured under standard conditions, however, a recent publication suggested that in vivo tissues have a broader cellular plasticity compared to organoids [44]. Interestingly, we observed a CD44+/LUM+ cell population in our CRC organoids that may represent an aggressive intermediate EMT cell state [45]. However, further studies are required for the more detailed analysis of these cells. 

The combination of MEKi and HSP90i is an effective targeted therapy in pancreas ductal adenocarcinoma (PDAC) without severe toxic side effects in mouse models [19]. In addition, a recent large-scale screen focused on compounds that are more active in CRC compared to normal colon organoids, and the authors identified the epigenetic inhibitor JQ1 as a promising molecule [21]. In addition, whereas normal colonic epithelial cells are dependent on BCL2, there is a shift in the intestinal adenoma phase to BCL-xL, thus, BCL-xL inhibitors are suggested to have a more profound effect in CRC [22]. In line with these data, whereas BCL-xLi induced apoptosis in mouse adenoma organoids, it had no effect in normal mouse intestinal organoids [22]. Thus, we selected drugs for testing that are expected to have a low toxicity effect on normal colonic epithelium. Although MEKi and JQ1 have already been used in combination on CRC patient-derived organoids [46], to our knowledge, we are the first to demonstrate that these compounds are highly effective in all combinations and they all act synergistically in CRC cells. However, we also found that the use of JQ1 enriched for the CD44^high^/LUM+ CRC cell population that may have an intermediate EMT phenotype. Interestingly, CAFs proved to be resistant to MEKi and highly sensitive to the BCL-xL inhibitor compared to organoids. Importantly, we found that the HSP90i negatively selected the tumor-promoting CD142^high^ fibroblasts. Of note, we also found the synergistic combination of BCLi and HSP90i in fibroblast-CRC organoid co-cultures. Since we did not study the direct cell contact between the different cell types, applying multicellular organoids better displaying the mixed CAF-cancer cell morphology of patient tumors would be an interesting option for further studies. Nevertheless, BCLi and HSP90i may be an effective combination to target both CRC cells and CAFs, and we suggest to further test them in pre-clinical models in vivo. 

In summary, we proved that CD142^high^ fibroblasts stimulate organoid formation and cell proliferation more efficiently compared to CD142^low^ cells in CRC by secreting a higher amount of HGF. TGFβ, a major inducer of CAFs, resulted in the accumulation of CD142^high^ fibroblasts that are in an active state. We also found that the combinations of novel compounds, BCLi, JQ1, HSP90i, and MEKi all act synergistically and with the exception of MEKi, they are also effective in CAFs. Since JQ1 selected for a CD44^high^/LUM+ phenotype in CRC cells, and HSP90i decreased the amount of CD142^high^ cells in CAF cultures, furthermore, both CRC cells and CAFs were sensitive to BCLi, thus, the combination of BCLi and HSP90i may represent a novel effective combination. Targeting both CRC cells and stromal fibroblasts would be desired to avoid tumor relapse. However, this strategy critically depends on fibroblast heterogeneity. Thus, our results on identifying a tumor-promoting CAF population and drug combinations targeting both tumor cells and these fibroblasts may form the basis of further pre-clinical studies.

## 4. Materials and Methods

### 4.1. Cell Cultures

Normal human colon fibroblasts (CCD-18Co, CRL-1459, ATCC, Manassas, VA, USA) were cultured in DMEM with 4500 g/L glucose (Gibco, Thermo Fisher Scientific, Waltham, MA, USA), 10% FBS (Biosera, Kansas, MO, USA), penicillin/streptomycin (Gibco) and glutamine (Merck, Darmstadt, Germany). Cells were washed with phosphate-buffered saline (PBS) three times and cultured in serum-free medium when applying ELISA (see below). Cell number was counted in a Burker chamber. We only used cells with low (<p8) passage number. Cell cultures used in our studies were negative for Mycoplasma contamination, tested with Hoechst staining. In some experiments, 5 nM coagulation factor VIIa (Invitrogen, Thermo Fisher Scientific, Waltham, MA, USA), 10 ng/mL TGFβ (Peprotech, London, UK), or 5 ng/mL IL1α (Peprotech) were used for 4 days in serum-free condition.

### 4.2. Isolation of Human Tumor Derived Fibroblasts

The Ethics Committee of the Medical Research Council of Hungary (ETT-TUKEB, No. 51323-4/2015/EKU) approved all experiments with human samples and informed consent was obtained from patients. Samples were collected at the Department of Oncosurgery, Uzsoki Hospital, Budapest, Hungary. Tumor tissues were cut into small pieces (<0.5 cm), and after washing with PBS three times, tissue pieces were incubated in a digestive mix (DMEM high glucose with 20% FBS, 125 µg/mL dispase type II (Invitrogen), 75 U/mL collagenase type II (Merck), 1 h) at 37 °C with shaking. Samples were then centrifuged at 300× *g* for 5 min to isolate single cells, they were washed twice in PBS and cultured in tissue culture plates (Eppendorf, Vienna, Austria) in DMEM high glucose, 10% FBS, penicillin/streptomycin and glutamine (fibroblast medium). In some experiments 5 nM factor VIIa, 10 ng/mL TGFβ, or 5 ng/mL IL1α were used for 4 days in serum-free medium. Patient data are shown in Table S2.

### 4.3. Human CRC Organoid Cultures and Quantification of Organoid Size

We used the CRC organoid lines published by our research group (see characterization of the organoids and patient data in Table S2 and [16,17]). Organoids were cultured in CRC medium composed of advanced DMEM/F12 (Gibco), 10 mM HEPES (Merck), glutamine, penicillin/streptomycin, B27 supplement (Gibco), 10 mM Nicotinamide (Merck), 1 mM N-Acetyl-Cysteine (Merck), 50 ng/mL EGF (Peprotech), 10 μM SB202190-monohydrochloride (Merck), and 500 nM A83-01 (Merck). To avoid anoikis, the Rho kinase inhibitor Y27632 (Merck) was also added for 3 days after passaging the organoids. Organoids were removed from the 3D matrix (Matrigel, Corning, New York, NY, USA) every 5–7 days mechanically, they were centrifuged at 600× *g* for 5 min, washed with PBS, and digested with TrypLE (Thermo Fisher, Waltham, MA, USA) until organoids were dissociated into smaller cell clusters. Samples were then washed, and embedded into 3D matrix again. When indicated, CRC organoid cells and CCD-18Co NCFs (5000–5000 cells) were mixed before embedding them into Matrigel as co-cultures. In some experiments 5 nM factor VIIa, 50 ng/mL HGF (Peprotech) or 50 ng/mL IL6 (Peprotech) were used for 4 days. 

Organoids were imaged and the diameter of the spherical organoids was measured with the ImageJ software (National Institutes of Health, USA). For each condition, we quantified more than 30 organoids for statistical evaluation (see below).

### 4.4. Collagen-Based Organoid Cultures 

To prepare 100 µL collagen I matrix, 60 µL distilled water, 10 µL 10× MEM (Gibco), and 30 µL collagen type I (from rat tail, Ibidi, Gräfelfing, Germany) were mixed, and the pH was set to 7.2 with 1 M NaOH. Organoids were isolated from Matrigel, washed twice with PBS, and embedded into collagen.

### 4.5. Flow Cytometry and Cell Sorting

Organoids or cells were dissociated into single cells by TrypLE and they were then suspended in FACS buffer (PBS with 1 mM EDTA, 25 mM HEPES, 1% bovine serum albumin (BSA)). Cells were labelled with primary antibodies and then with secondary antibodies for 20 min. We measured 10,000 events with a Cytoflex (Beckman Coulter, Brea, CA, USA) instrument, or cell subpopulations were sorted by a Sony SH800S cell sorter (Sony Biotechnology, Bothell, WA, USA). Identical cell numbers were then applied in the same culturing experiment to receive comparable data. Antibodies are listed in Table S3.

### 4.6. Immunocytochemistry and Whole-Mount Immunostaining

Cells were fixed in 4% paraformaldehyde (PFA) for 20 min, washed with PBS, and then blocked and permeabilized in blocking buffer (PBS + 5% FBS + 0.2% BSA + 0.3% Triton X-100). Primary antibodies were applied at 4 °C overnight and then secondary antibodies for 2 h at room temperature (RT) (all diluted in blocking buffer). Samples were covered with ProLong Diamond antifade mounting medium with DAPI (Thermo Fisher Scientific). 

Organoids were cultured in 8-well chamber slides (BD Biosciences, East Rutherford, NJ, USA), fixed in 4% PFA for 40 min, washed with PBS three times, and blocked and permeabilized with WBB whole-mount blocking buffer (PBS + 5% FBS + 0.2% BSA + 0.3% Triton X-100) for 2 h. Samples were incubated with primary antibodies at 4 °C overnight in WBB, and labelled with secondary antibodies for 2 h at RT. Organoids were then mounted with ProLong Diamond antifade mountant containing DAPI (Thermo Fisher Scientific). Confocal images were taken with a Leica TCS SP8 microscope, and we used the ImageJ software for analysis and quantification. Antibodies are listed in Table S3.

### 4.7. Immunofluorescent Staining of Paraffin Embedded Sections

PFA-fixed and paraffin-embedded sections were deparaffinized and rehydrated according to standard methods. Sections were then boiled in Tris-EDTA high-pH buffer (10 mM Tris base, 1 mM EDTA solution, 0.05% Tween-20 in distilled water, pH = 9.0) for 15 min and allowed to cool to RT for 20 min. After washing, sections were blocked in WBB, primary antibodies were applied at 4 °C overnight, and secondary antibodies were applied for 2 h at room temperature (all in WBB). Samples were covered with ProLong Diamond antifade mountant containing DAPI (Thermo Fisher Scientific).

### 4.8. ELISA

Sorted CD142^low^ and CD142^high^ fibroblasts (100,000 cells/well, 48-well plate) were cultured for 2 days, FBS was removed from the medium and cells were further cultured for 3 days. Cell debris was removed from the conditioned medium by centrifugation (300× *g*, 5 min), and HGF was detected with ELISA (Bio-Techne, Minneapolis, MN, USA, DHG00B) according to the manufacturer’s protocol. We measured the OD values of the plates on a HiPo MPP-96 Microplate Photometer (Biosan, Riga, Latvia).

### 4.9. RNA Isolation and Expression Analysis

Total RNA was isolated with the miRNEasy Micro Kit (Qiagen, Hilden, Germany). In some experiments, cells were directly sorted into Qiazol (Qiagen). RNA concentration was determined with a NanoDrop instrument (Thermo Fisher). We used the Sensi-FAST cDNA Synthesis Kit (Bioline, Meridian Bioscience, London, UK) to reverse-transcribe 150 ng RNA (in 20 µL final volume), and quantitative PCR reactions were carried out with the SensiFAST SYBR NO-ROX Kit (Bioline) on a BioRad (Hercules, CA, USA) CFX384 Touch real-time PCR instrument (384-well format, 5 µL/well volume) using the SybrGreen method. Results were evaluated according to this equation: relative expression level = 2−ΔCt, where ΔCt = Ct(gene of interest)—Ct(housekeeping gene). The primers are listed in Table S4.

### 4.10. Viability Assay

To determine the number of viable cells, we used the CellTiter-Glo 3D Cell Viability Assay (Promega, Madison, WI, USA) according to the manufacturer’s protocol in a 96-well format, and we scanned the plate with a Fluoroskan FL (Thermo Fisher Scientific) instrument. Then, 5000 organoid cells or fibroblasts were embedded into 6 μL 3D matrix, cultured for 4 days, and then treated with the compounds for 6 days. In some experiments, CRC organoid cells and CCD-18Co NCFs (5000–5000 cells) were mixed before embedding them into Matrigel as co-cultures. 5-fluorouracil, irinotecan, trametinib (GSK1120212), PU-H71, A-1155463 and (+)-JQ1 were purchased from Selleck Chemicals (Breda, The Netherlands). All compounds were dissolved in DMSO to prepare a stock solution according to the manufacturer’s protocol, and they were used in the concentration range of 20,000 and 3 nM with 3-fold dilution series. Viability results were evaluated with the following equation: well viability % = (well value—average positive control)/(average vehicle control—average positive control) × 100. DMSO served as vehicle control, 5 μM staurosporine (MedChemExpress, Monmouth Junction, NJ, USA) as positive control [47]. 

IC50 value (half-maximal inhibitory concentration) was calculated using the AAT Bioquest IC50 calculator web tool (https://www.aatbio.com/tools/ic50-calculator, accessed on 20 June 2023), and synergy was determined using the Chou-Talalay method with CompuSyn software (https://www.combosyn.com/, accessed on 20 June 2023). Synergistic and antagonistic effects were determined according to [19,48]. Briefly, the calculated combination index (CI) value <0.75 and >1.25 indicated synergistic and antagonistic effects, respectively.

### 4.11. Bioinformatical and Statistical Analysis

Intensity and quantity data for CRC from the Human Protein Atlas database (https://www.proteinatlas.org/, accessed on 28 April 2023) were analyzed for CD142 (F3) and PDPN immunostaining. For statistical analysis of our experimental data, Student’s paired or unpaired *t*-tests, or Mann–Whitney U-tests were applied with * *p* < 0.05, ** *p* < 0.01, and *** *p* < 0.005 significance levels. Microsoft Excel (Redmond, WA, USA), IBM SPSS version 25 (Armonk, NY, USA), and GraphPad software (Boston, MA, USA) were used for statistical evaluation. Mean + SD or median, 25th, and 75th percentiles for the box plots are shown.

## Data Availability

Data or material used in this study are available from the corresponding author upon reasonable request.

## Supplementary Materials

The following supporting information can be downloaded at: https://www.mdpi.com/article/10.3390/ijms241411585/s1. References [16,17] are cited in the supplementary materials
