# Supplementary material for: High CD142 Level Marks Tumor-Promoting Fibroblasts with Targeting Potential in Colorectal Cancer
Source: Int J Mol Sci. 2023 Jul 18;24(14):11585. doi: 10.3390/ijms241411585 (PMC10381019; doi:10.3390/ijms241411585)
Supplement: Supplementary file 1 [file ijms-24-11585-s001.zip › ijms-2403929-supplementary.pdf]

# High CD142 level marks tumor promoting fibroblasts with targeting potential in colorectal cancer

András Áron Soós[1], Andrea Kelemen[1], Adrián Orosz[1], Zsuzsanna Szvicsek[1], Tamás Tölgyes[2], Kristóf Dede[2], Attila Bursics[2], Zoltán Wiener[1]

[1]Department of Genetics, Cell and Immunobiology, Semmelweis University, Budapest, Hungary

[2]Uzsoki Teaching Hospital, Budapest, Hungary

## Supplementary Tables

| Compounds | Org1 | Org2 | Org3 | Org4 | NCFs | | | CAF1 | CAF2 | CAF3 | CAF4 |
|---|---|---|---|---|---|---|---|---|---|---|---|
| MEKi (nM) | 19 | 18 | 14 | 28 | 309 | 397 | 273 | 193 | 188 | 151 | 127 |
| HSP90i (nM) | 306 | 349 | 402 | 302 | 380 | 503 | 326 | 53 | 225 | 132 | 119 |
| JQ1 (nM) | 669 | 1196 | 1824 | 2050 | 203 | 130 | 227 | 134 | 202 | 146 | 131 |
| BCLi (nM) | 1336 | 2565 | 738 | 1344 | 237 | 128 | 368 | 451 | 689 | 358 | 328 |

| Compounds | Org1 | Org2 | Org3 | Org4 | NCFs | | |
|---|---|---|---|---|---|---|---|
| MEKi+JQ1 (nM) | 131 | 274 | 180 | 100 | 305 | 255 | 237 |
| MEKi+BCLi (nM) | 36 | 70 | 71 | 140 | 860 | 571 | 832 |
| HSP90i+JQ1 (nM) | 196 | 691 | 194 | 770 | 1271 | 1372 | 1364 |
| HSP90i+BCLi (nM) | 54 | 280 | 68 | 793 | 356 | 453 | 417 |
| MEKi+HSP90i (nM) | 33 | 72 | 71 | 104 | 258 | 253 | 218 |
| JQ1+BCLi (nM) | 193 | 138 | 72 | 257 | 35 | 36 | 31 |
| 5FU (nM) | 18073 | 23603 | 47720 | 36287 | | | |
| Irino (nM) | 649 | 486 | 554 | 1025 | | | |
| 5FU+Irino (nM) | 104340 | 102170 | 79700 | 152560 | | | |

**Table S1.** IC50 values for the used compounds and combinations in CRC organoids and colon fibroblasts.

**A)**

| Sample | Gender | Age | Tumor | Differentiation grading | Stage | Nutlin sensitivity | EGF dependency |
|---|---|---|---|---|---|---|---|
| CRC1 | F | 63 | colon adenocarcinoma | Grade 2 | T3N0M0 | no | yes |
| CRC2 | M | 74 | colon adenocarcinoma | Grade 2 | T3N0M0 | no | yes |
| CRC3 | M | 75 | colon adenocarcinoma | Grade 2 | T3N2aM0 | no | yes |
| CRC4 | M | 74 | colon adenocarcinoma | Grade 2 | T3N1M0 | yes | yes |

**B)**

| Sample | Gender | Age | Tumor | Differentiation grading | Stage |
|---|---|---|---|---|---|
| CRC-CAF1 | F | 39 | Colon adenocarcinoma | Grade 2 | T3N2bM1a |
| CRC-CAF2 | F | 37 | Colon adenocarcinoma | Grade 2 | T3N1aM0 |

| | | | | | |
|---|---|---|---|---|---|
| CRC-CAF3 | F | 67 | Colon adenocarcinoma | Grade 2 | T3N0M0 |
| CRC-CAF4 | F | 56 | Colon adenocarcinoma | Grade 2 | T3N1aM0 |
| CRC-CAF5 | M | 66 | Colon adenocarcinoma | Grade 2 | T3N0M0 |
| CRC-CAF6 | M | 68 | Colon adenocarcinoma | Grade 2 | T3N0M0 |
| CRC-CAF7 | F | 55 | Colon adenocarcinoma | Grade 2 | T3N0M0 |
| CRC-CAF8 | M | 71 | Colon adenocarcinoma | Grade 2 | T3N1bM0 |
| CRC-CAF9 | F | 79 | Colon adenocarcinoma | Grade 2 | T4bN0M0 |
| CRC-CAF10 | M | 77 | Colon adenocarcinoma | Grade 2 | T3N0M0 |
| CRC-CAF11 | M | 57 | Colon adenocarcinoma | Grade 2 | T3N1bM0 |
| CRC-CAF12 | F | 81 | Colon adenocarcinoma | Grade 2 | T3N1bM0 |
| CRC-CAF13 | F | 47 | Colon adenocarcinoma | Grade 2 | T4aN1aM0 |
| CRC-CAF14 | F | 56 | Colon adenocarcinoma | Grade 2 | T3N1bM0 |
| CRC-CAF15 | M | 70 | Rectal adenocarcinoma | Grade 2 | T3N0M0 |
| CRC-CAF16 | F | 65 | Colon adenocarcinoma | Grade 3 | T3N0M0 |
| CRC-CAF17 | M | 62 | Colon adenocarcinoma | Grade 2 | T3N0M0 |
| CRC-CAF18 | M | 82 | Colon adenocarcinoma | Grade 2 | T1N0M0 |
| CRC-CAF19 | F | 70 | Colon adenocarcinoma | Grade 3 | T3N0M0 |
| CRC-CAF20 | F | 69 | Colon adenocarcinoma | Grade 2 | T3N1bM0 |
| CRC-CAF21 | F | 46 | Colon adenocarcinoma | Grade 2 | T4bN1cM0 |

**Table S2.** Clinical data. A) Data of patient derived organoids. Nutlin resistance indicates the lack of wild-type *TP53* and EGF dependence marks the lack of mutations in the EGF signalling pathway (e.g. KRAS and BRAF). For further characterization of the organoid lines see [1, 2]. B) Clinical data of patients providing fibroblast cells.

| Antibody | Producer | Catalog/Clone number |
|---|---|---|
| human CD142-PE | BioLegend | #365204 |
| human CD44-PE | BD Pharmingen | #555479 |
| human podoplanin-APC | BioLegend | #337022 |
| human CD142 | BioLegend | #365202 |
| human HGF | R&D Systems | AF-294-NA |
| human KI67 | eBioscience | 14-5699-82 |
| human lumican | Abcam | ab168348 |
| human phospho-p42/44 | Cell Signaling | #9101 |
| human phospho-S6 | Cell Signaling | #2211 |
| human vimentin | R&D Systems | MAB2105 |
| human αSMA | Sigma | A5228 |
| human EpCAM | R&D Systems | AF960 |
| human active caspase-3 | R&D Systems | AF835 |
| goat IgG Alexa 488 | Invitrogen | A21467 |
| mouse IgG Alexa 488 | Invitrogen | A21202 |
| rabbit IgG Alexa 568 | Invitrogen | A11011 |
| rabbit IgG Alexa 750 | Invitrogen | A21039 |
| rat IgG Alexa 568 | Invitrogen | A11077 |

**Table S3.** Antibodies used in our studies.

| Gene | Primer1 | Primer2 |
|---|---|---|
| ACTA2 | CTGACCCTGAAGTACCCGAT | GTCATTTTCTCCCGGTTGGC |
| AREG | CCTACTCTGGGAAGCGTGAA | AGTAGTCATAGTCGGCTCCC |
| AXIN2 | CTGGCTATGTCTTTGCACCA | CTTCACACTGCGATGCATTT |
| BAD1 | CCGGAGGATGAGTGACGAGT | AAGTTCCGATCCCACCAGGA |
| BCL2 | ATGTGTGTGGAGAGCGTCAA | GGGCCGTACAGTTCCACAAA |
| BCL-XL | TGCAGGTATTGGTGAGTCGG | CTGAAGAGTGAGCCCAGCAG |
| BID | CCAGAACCTACGCACCTACG | ACCACATCGAGCTTTAGCCA |
| BIM | TCATCGCGGTATTCGGTTCG | ACCTTCTCGGTCACACTCAG |
| BTC | ACTGCATCAAAGGGAGATGC | CATGTGCAGACACCGATGA |
| CDH1 | GGATGTGCTGGATGTGAATG | AGCCAGTTGGCAGTGTCTCT |
| COL1A1 | AACGATGGTGCTAAGGGTGA | CCATCTTTGCCAGGAGAGCC |
| CTGF | CGAAGCTGACCTGGAAGAGA | CCGTCGGTACATACTCCACA |
| CXCL1 | GTCCGTGGCCACTGAACT | TTTCCGCCCATTCTTGAGTG |
| CSF3 | GGACACTCTCTGGGCATCC | CAGCTGCAGTGTGTCCAAG |
| EGF | CAGATCTCGATGGTGTGGGA | CCTCCATCATAATCACAGGAGC |
| EpCAM | CTGGCCGTAAACTGCTTTGT | CATCATTGTTCTGGAGGGCC |
| EREG | TCCATCTTCTACAGGCAGTCC | TGCACTGTCCATGCAAACAA |
| FAP | GGAAATGAGCTTCCTCGTCC | GGTGGATCTCCTGGTCTTTG |
| GAPDH | GGGTGTGAACCATGAGAAGT | CAGTGATGGCATGGACTGTG |
| HBEGF | TTATCCTCCAAGCCACAAGC | CCCATGACACCTCTCTCCAT |
| HGF | CAAGCAATCCAGAGGTACGC | TGGTGTCTGATGATCCCAGC |
| IL11 | GACAAATTCCCAGCTGACGG | CGCAGGTAGGACAGTAGGT |
| IL6 | ATTCCAAAGATGTAGCCGCC | AGTGCCTCTTTGCTGCTTTC |
| LUM | CCTGGTTGAGCTGGATCTGT | GTAGGATAATGGCCCCAGGA |
| MCL1 | GACGAGTTGTACCGGCAGTC | TGATGTCCAGTTTCCGAAGCA |
| MYC | TCCTCGGATTCTCTGCTCTC | CTCTGACCTTTTGCCAGGAG |
| TGFA | CCTGCCTAGTCTGCGTCTTT | CCAACACAATACCCAGAGCG |
| VEGFA | AAGGAGGAGGGCAGAATCAT | CACACAGGATGGCTTGAAGA |
| VIM | GGTACTCGCATTCTCCACCT | CTCAATGTCAAGGGCCATCT |
| WNT4 | ACAGTCGTTTGTGGATGTGC | CCAGCACGTCTTTACCTCAC |
| WNT5A | CAAGGGCTCCTACGAGAGTG | CTTCTCCTTCAGGGCATCAC |
| WNT5B | TTTGGGAGAGTCATGCAGAT | TAGCCGTACTCCACGTTGTC |
| ZEB1 | GCTGACTGTGAAGGTGTACC | ACATCCTGCTTCATCTGCCT |

**Table S4.** Primers used in our studies.

**Supplementary references**

1. Szvicsek Z, Oszvald A, Szabo L et al. Extracellular vesicle release from intestinal organoids is modulated by Apc mutation and other colorectal cancer progression factors. Cell Mol Life Sci. 2019;76:2463-2476.
2. Kelemen A, Carmi I, Oszvald A et al. IFITM1 expression determines extracellular vesicle uptake in colorectal cancer. Cell Mol Life Sci. 2021;78:7009-7024.

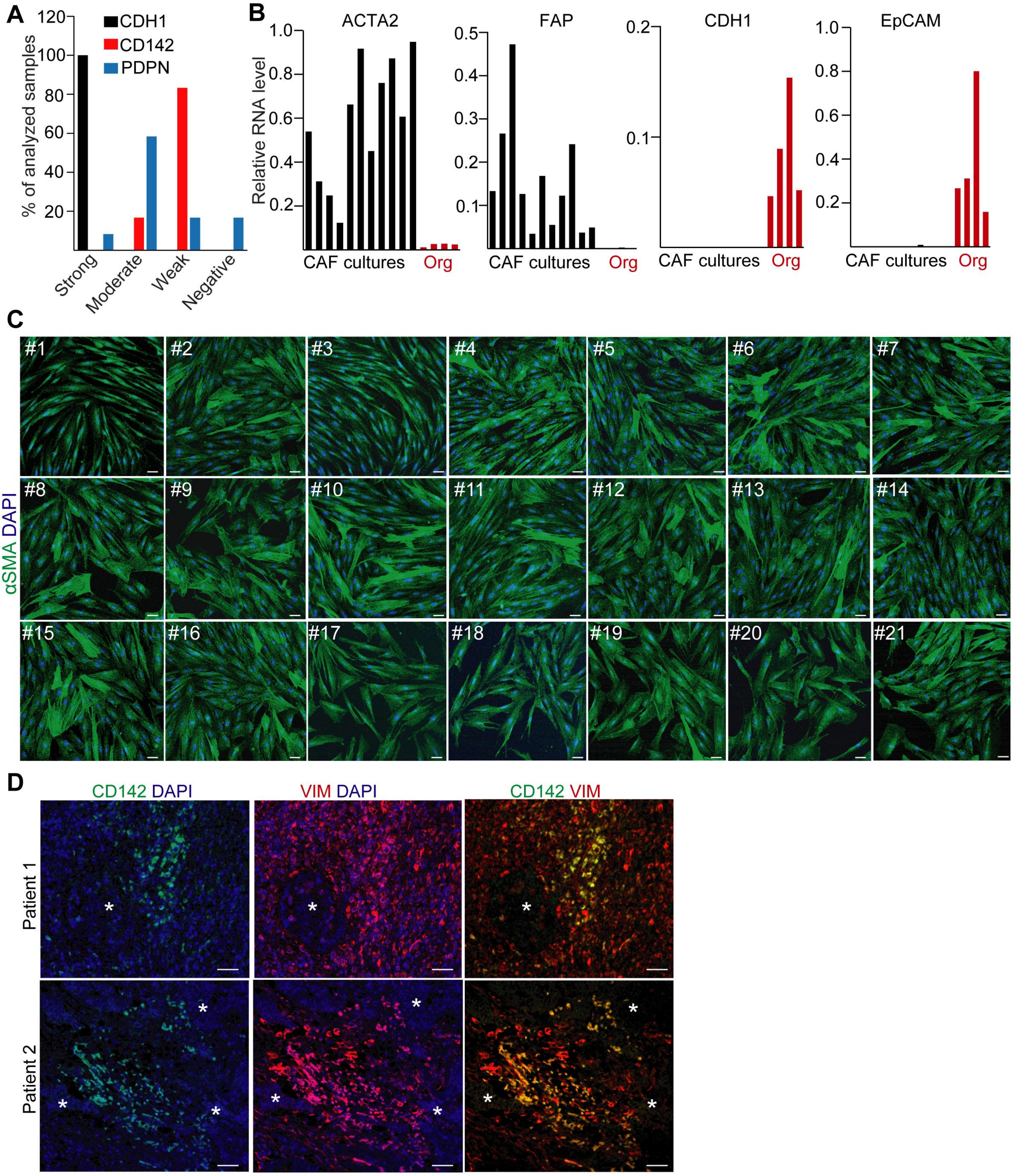


**Figure S1. A subpopulation of the stromal cells expresses CD142 in CRC tissue sections.** A) The percentage of samples with CRC tumor cells showing strong, moderate, weak, or no immunostaining for CD142 or podoplanin (PDPN) (analysis of data from Protein Atlas, www.proteinatlas.org). CDH1 (E-cadherin) was used as a positive control. B) The relative RNA level of mesenchymal (*ACTA2* encoding for αSMA, and *FAP*) and epithelial (*CDH1, EpCAM*) markers in CAF cultures and CRC organoid lines (RT-qPCR). Results were normalized to *GAPDH* housekeeping. C) α-smooth muscle actin (αSMA) immunostaining of the CAF lines. Note that the majority of the cells are positive for this marker. DAPI marks the nuclei. D) Detecting CD142 and the mesenchymal marker vimetin (VIM) in CRC patient-derived tumor tissue sections. Scale bars: 40 µm (C) or 100 µm (D). Asterisks mark the tumor tissues.

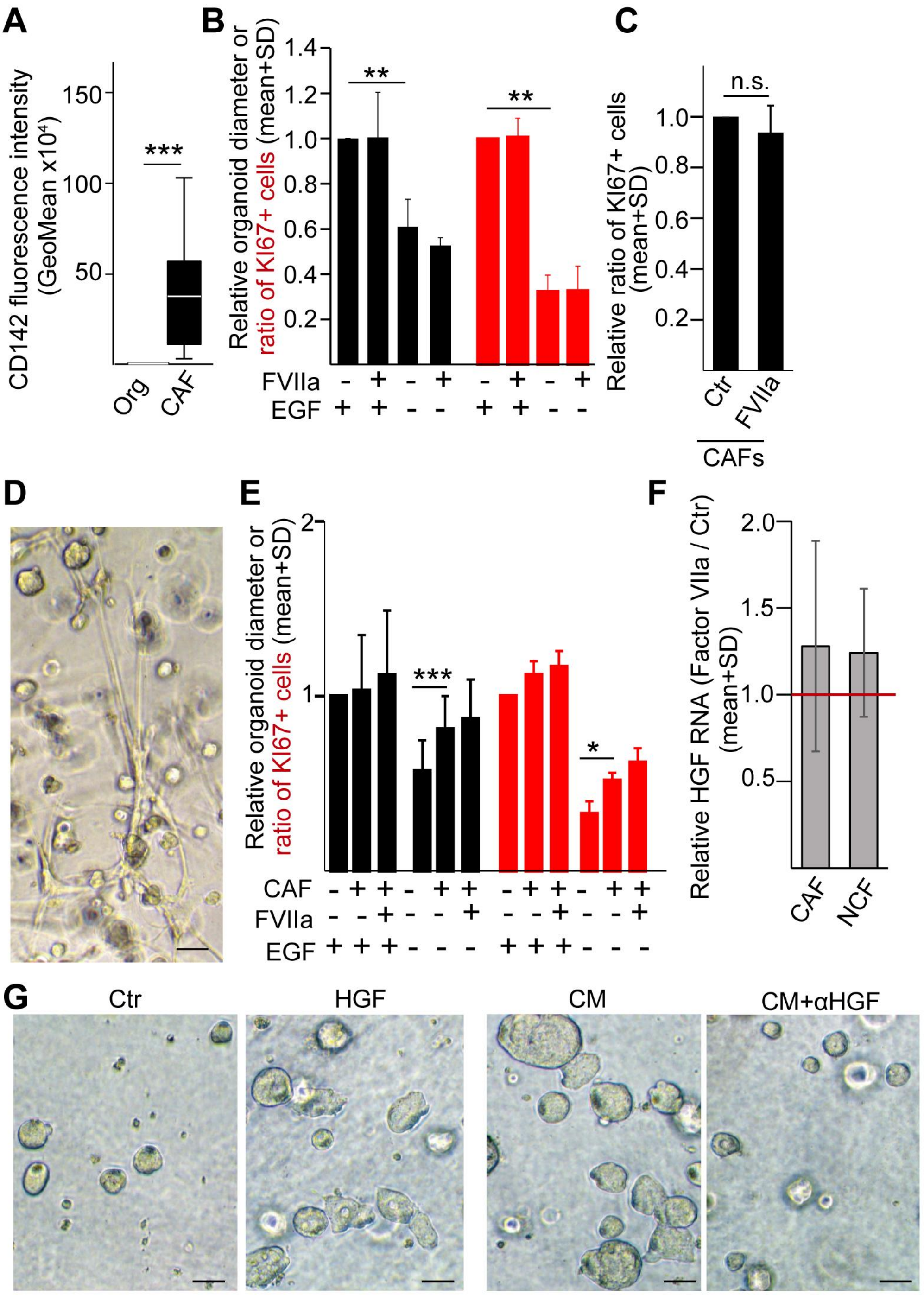


**Figure S2. Factor VIIa has no effect on either fibroblasts or CRC cells.** A) CD142 cell surface level (geometric mean, GeoMean) from CRC organoid cells or CAFs (flow cytometry, n=8 for CRC and n=12 for CAFs. For CRC, four organoid lines were measured twice). B) Organoid diameters and the ratio of proliferating KI67+ CRC cells in the presence or absence of EGF and/or factor VIIa. Values were compared to the control (with EGF and without factor VIIa, n=4). 5 nM factor VIIa was applied for 4 days. C) Cell proliferation intensity in CAF cultures with/without 5 nM factor VIIa (4 days, n=5). D) Light microscopy of the co-culture of CRC organoids and CAFs (scale bar: 20 µm). E) Organoid diameter and ratio of KI67+ cells of CRC organoids in the absence or presence of factor VIIa, EGF or CAFs. Treatments were carried out for 4 days (n=4). F) Relative HGF RNA levels in the factor VIIa treated fibroblasts. Data were normalized to housekeeping control and then compared to the untreated controls marked with red line (RT-qPCR, n=4). G) Representative light microscopy images of CRC organoids in the presence of the indicated treatments (HGF 50 ng/mL, αHGF 5µg/mL for 4 days). Conditioned media (CM) from NCFs were used after 72 hours of culturing (scale bars: 100 µm). Mann-Whitney U-test (A) or paired t-test (B-F) were applied with n.s.: $p>0.05$, *$p<0.05$, **$p<0.01$ and ***$p<0.005$.

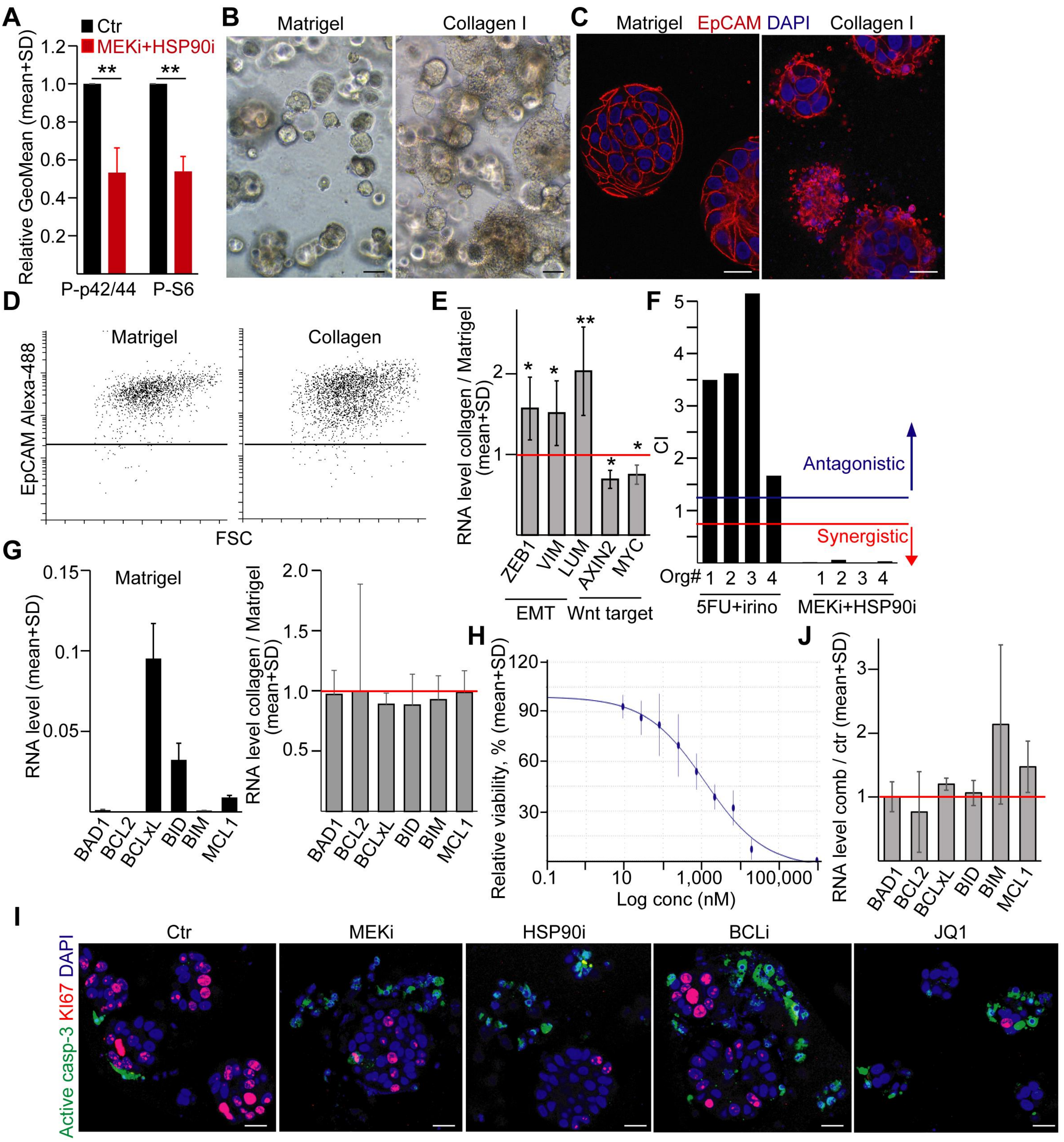


**Figure S3. Collagen-I does not modify the expression level of BCL2 family members and the effect of compound combinations.** A) Phospho-S6 and phospho-p42/p44 levels in CRC organoid cells with or without MEKi and HSP90i (n=4 from four organoid lines). B) The morphology of CRC organoids in Matrigel and in collagen-I (light microscopy). Note the invading cells in collagen. C) EpCAM immunostaining and confocal microscopy images of organoids in Matrigel or collagen. D) Flow cytometry of CRC organoid cells for EpCAM. E) Changes of the RNA level of the indicated genes in collagen-I compared to Matrigel (n=4). RT-qPCR results were normalized to the housekeeping GAPDH, and normalized data from collagen-I were compared to Matrigel. The red line indicates no change (ratio=1). F) Synergistic (combination index, CI<0.75, red line) or antagonistic (CI>1.25, blue line) effects of the indicated compound combinations in CRC organoids cultured in collagen-I. G) RNA levels of BCL2 family members in CRC organoids (n=4, RT-qPCR, left panel) in Matrigel and changes in the relative RNA levels when culturing them in collagen (right panel, n=4 from four organoid lines). Data were normalized to GAPDH. H) Organoid viability in the presence of the BCLi. I) Immunostaining for the indicated proteins (confocal microscopy). Note that compounds were applied at the IC50 concentration for 2 days. J) Relative RNA level of the indicated genes when organoids were treated with BCLi and HSP90i (combination treatment) compared to the control (RT-qPCR, n=4). Values normalized to GAPDH were compared. Paired t-tests were applied with *p<0.05 and **p<0.01. Scale bars: 100 µm (B), 20 µm (C, I).
